# The Case of XPD: Sometimes Two Different Mutant Genes Are Better than One

**DOI:** 10.1371/journal.pbio.0040347

**Published:** 2006-10-03

**Authors:** Liza Gross

Rare inherited disorders have long provided a unique window on the genetic basis of disease. Individuals inherit two copies of a gene; one from each parent. In a recessive disorder, if one copy is defective (a mutant allele), the alternate copy is usually sufficient to maintain normal function of the encoded protein. If an individual inherits two defective copies, protein function is disrupted, leading to disease. Often, two different mutant alleles of the same gene are present in one person, a phenomenon called compound heterozygosity. Whether these still cause disease depends on the gene in question.

The potential for recessive genes to interact has rarely been studied in human disease largely because distinguishing the effects of environment and genetic background from “biallelic” effects is very difficult in humans. In a new study, Jaan-Olle Andressoo, James Mitchell, and colleagues circumvent this problem by using a compound heterozygous mouse model of a severe human syndrome called trichothiodystrophy (TTD) that allowed them to link physical traits (or phenotype) to specific combinations of mutant alleles.

TTD belongs to a class of rare, clinically distinct XPD-related recessive disorders. XPD, a DNA-unwinding enzyme, is essential for both gene transcription and DNA repair of sun-induced damage as a component of the transcription/repair factor IIH (TFIIH) complex. In addition to TTD, XPD mutations cause xeroderma pigmentosum (XP) and Cockayne syndrome (CS). XP results in dramatically elevated cancer risk from extreme sun sensitivity—though, surprisingly, sun sensitivity does not necessarily cause skin cancer—and, in severe cases, primary neurodegeneration. Neither CS nor TTD increase cancer risk but lead to accelerated aging, reduced stature, and degeneration of the nerves’ protective myelin sheath. TTD also causes scaly skin and brittle hair. As its name implies, the even rarer XP combined with CS (XPCS) combines cancer predisposition with neurodevelopmental problems.

In the current model, which Andressoo et al. refer to as the “monoallelic” paradigm of XPD-related disease, “causative” mutations are linked to a specific XPD syndrome. Mutations that aren’t linked to a particular disorder are considered biologically inactive, or null. But the appearance of patients with causative mutations and a rare combination of TTD and XP symptoms has revealed the limitations of this paradigm. To explore the potential role of what the researchers call “biallelic effects” in human recessive disorders, Andressoo et al. asked whether different allele combinations of the enzyme XPD influence the diverse phenotypes associated with XPD-related recessive disorders. They discovered that combinations of XPD recessive alleles produced a variety of biallelic effects, from alleviating the severity of various disease symptoms to improving the function of the interacting genes.

In addition to their existing TTD mouse model (with the causative XPDR^722W^ mutation), Andressoo et al. “knocked in” a mutation found in an XPCS “hemizygote” patient (XPD^G602D^), who had only one copy of the gene, suggesting that mice carrying two copies of this allele (homozygotes) should live. However, no *Xpd^G602D^* homozygous mutants lived, and the *Xpd^G602D^* allele was designated *Xpd^†XPCS^* or “lethal.” Lethality was likely caused by the reduced expression of the mutant allele rather than by the mutation itself, the researchers concluded, because they found in a separate study that normalization of XPD^G602D^ expression levels leads to viable homozygous animals. Thus, the XPD^G602D^ protein is likely biologically active but its reduced expression in homozygous *Xpd^†XPCS^* animals causes lethality. Knocking in an *Xpd* mutation (encoding XPD^R683W^) associated with XP was also homozygous lethal (and designated *Xpd^†XP^)*, probably for the same reason.

**Figure:**
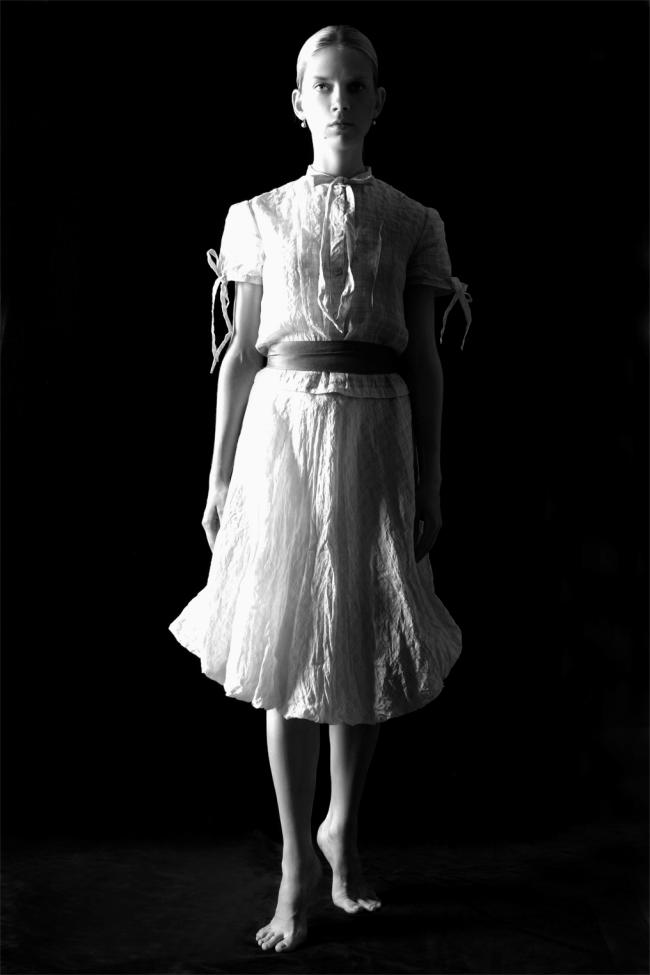
The surprising rescue of disease symptoms in mice suggests new thinking about recessive disorders in humans. Potential phenotypic consequences of compound heterozygosity are represented by a continuum from dark (disease) to light (health). (Photo: L. Kallasvee/J.O. Andressoo; Model: Reet Aus)

To see if these homozygous lethal alleles might interact with a different disease-causing allele, the researchers generated compound heterozygous mice with the *Xpd* homozygous lethal allele *(Xpd^†XPCS^)* and a TTD-causing allele *(Xpd^TTD^)*. Multiple skin, hair, and aging-related features of TTD were far less severe in the compound heterozygous animals than in animals carrying two copies of the TTD-causing allele. Beyond ameliorating these classic TTD symptoms, the homozygous lethal allele alleviated anemia and developmental delay and also extended lifespan in the compound heterozygotes. Similarly, generating compound heterozygotes from the homozygous lethal XP allele *(Xpd^†XP^)* and the TTD-causing allele attenuated the TTD-related skin and weight-loss symptoms. The researchers propose that, due to the low expression levels, the lethal alleles, when homozygous, lead to a transcriptional defect that proves fatal. But when either allele is combined with the TTD-causing allele, the latter steps in to perform the transcription task early enough to prevent embryonic lethality. Then, later, as the skin, hair, and blood cells develop, the lethal alleles recover the deficiencies of the TTD allele.

Combining one of the homozygous lethal alleles (*Xpd^†XPCS^* or *Xpd^†XP^)* with a TTD-causing allele also allowed the normally sun-sensitive XPCS and XP cells to better survive ultraviolet light. This finding suggests that interactions between the alleles produce an effect—resistance to sunlight—that neither has on its own, a phenomenon called “interallelic complementation.” The researchers suspect that complementation occurs as different XPD molecules are plugged into the TFIIH complex at the site of DNA damage.

These results suggest that even though presumed-“null” alleles can’t execute their transcription task, they may still influence disease outcome in compound heterozygous patients, as they have in the mouse model. The evidence that both alleles can contribute to disease phenotype, the researchers conclude, also suggests that it’s time to adopt a biallelic paradigm for compound heterozygous patients with XPD-related disorders.

